# Using longitudinal data to understand nutrition and health interactions in rural Gambia

**DOI:** 10.1080/03014460.2020.1718207

**Published:** 2020-05-20

**Authors:** Sophie E. Moore

**Affiliations:** a Department of Women & Children's Health, King's College, London, UK;; b MRC Unit The Gambia at the London School of Hygiene and Tropical Medicine, Fajara, The Gambia

**Keywords:** Cohorts, DOHaD, The Gambia, Africa

## Abstract

**Context**: Population-based cohort studies have been pivotal in establishing a number of nutrition-health interactions, especially in high-income settings. Less research is available from low- and middle-income countries due to the lack of detailed longitudinal data.

**Objective**: To describe the use of prospectively collected longitudinal data from the rural West Kiang region of The Gambia to explore nutrition-health interactions in a rural sub-Saharan African context.

**Methods**: Demographic records initiated in 1947, coupled with data on maternal and child health, have been used to explore nutrition-health relationships.

**Results**: An analysis of the longitudinal demographic data demonstrated a highly significant association between season of birth and infection-related adult mortality in this context. Additionally, using routine data on childhood anthropometry, it has been shown that, despite a significant decline in child undernutrition, rates remain unacceptably high, likely reflecting the very high socio-economic threshold required to eliminate undernutrition.

**Conclusion**: The foresight to establish demographic data collection over seventy years ago has supported a wealth of novel research within a traditional African context. The availability of detailed clinical records on maternal and child health is helping to unravel the factors driving child undernutrition in rural Africa, and to identify targets for interventions to improve health in this context.

## Introduction

Population-based cohort studies have contributed to great advances in our understanding of human health. For example, within the UK context, longitudinal cohorts including the 1958 National Child Development Study (Power and Elliott 2006), the 1970 British Cohort Study (Elliott and Shepherd 2006) and, more recently, the Avon Longitudinal Study of Parents and Children (ALSPAC) (Fraser et al. 2013) and Born in Bradford (BiB) studies (Wright et al. 2013) are robust examples of how long-term investments in prospective birth cohorts have yielded rich outputs both with respect to scientific knowledge and policy support. In addition, and alongside retrospective birth cohorts such as the Hertfordshire cohort in the UK (Syddall et al. 2005), these longitudinal birth cohorts have been pivotal in underpinning the research now framed within the “Developmental Origins of Health and Disease” (DOHaD) hypothesis.

DOHaD argues that the early-life environment has lifelong effects on human health (Hanson 2015) and much of the evidence in support of this hypothesis comes from cohort studies, where early life records are linked to later life health outcomes. Although a lot of the early focus of DOHaD came from cohort studies in high income settings, the importance of well-maintained cohorts from low-and middle-income countries (LMICs) has become a key feature of this field. As an example, the COHORTS (Consortium of Health-Orientated Research in Transitioning Societies) consortium, brings together data from transitioning societies in India, Brazil, Guatemala, South Africa and the Philippines (Richter et al. 2012) and has made a number of important contributions to our understanding of links between early exposures and life course health risks (e.g. Martorell et al. 2010; Stein et al. 2010; Fall et al. 2015). In sub-Saharan Africa, however, far less research is available, largely due to the lack of detailed longitudinal datasets in these contexts (Norris et al. 2017).

One exception to this is for the rural population from the West Kiang region of The Gambia. For almost seven decades, detailed demographic and health related data have been rigorously collected for this population, contributing to a rich database on several overlapping cohorts. In this paper, the history of the “Keneba cohorts” is described, and some of the key outputs from this wealth of demographic and health-related data reviewed.

## Methods

The UK Medical Research Council (MRC) has invested in a health-related research programme in The Gambia since 1947 when the WWII British Army Hospital in Fajara was handed over to the MRC and a permanent research station established. The MRC’s main operational base remains in Fajara, which is located on the coast, approximately 9 miles from the capital Banjul.

The population surveyed through the Keneba cohorts comprises residents of the West Kiang region of The Gambia, a rural region located approximately 100 miles from Banjul. West Kiang consists of approximately 750 km^2^ of savannah and farmland, bounded on 3 sides by the River Gambia and its tributaries. This predominantly Muslim community has typically relied on subsistence farming agriculture, with some petty trading. In this region food availability fluctuates widely across the year and the wet season, lasting from July to October, is a “lean” period when stored staple foods from the previous year’s harvest are nearly depleted and adults are engaged in physically intense agricultural activities. Full details about the region have been reported elsewhere (Moore 2016; Hennig et al. 2017). The data that now constitutes the Keneba cohorts have broadly been collected across three periods of time; 1949 to 1976; 1976 to 2004; and 2004 to date. Details of the data collected during each phase are given below.

### Phase 1, 1949 to 1976

In October 1949, Dr Ian McGregor joined the Nutrition Research cadre at the MRC’s Fajara station in The Gambia with the task to investigate the possible contributory role of parasitic infections on protein-calorie malnutrition. Based on its remoteness and lack of medical services of any kind, together with a high incidence of splenic enlargement and anaemia in children under 10 years of age, McGregor selected the village of Keneba with the nearby villages of Manduar, Kantong Kunda and Jali for surveillance. These villages were then visited annually by McGregor and his team for the collection of detailed records of growth, morbidity and mortality. In between the annual surveys, a system for routinely collecting demographic data in each village was established. At this time, the population of Keneba was approximately 700 in number (Rayco-Solon et al. 2004). During this period, McGregor led a research programme on malaria, with a specific interest in acquired malarial immunity and published a number of seminal papers on this topic (McGregor et al. 1983; McGregor 1985). However, of relevance to the current review, it was his foresight to introduce meticulous record keeping through both the annual health surveys and the regular demographic surveillance that must be acknowledged. These records have contributed to what is now the longest demographic surveillance of any population in rural, sub-Saharan Africa and have been pivotal in forming the basis of the health and nutrition related research reviewed in the latter part of this paper.

### Phase 2, 1976 to 2004

From 1974, and as a consequence of the enforced closure of the MRC Child Nutrition Unit in Uganda, the MRC Dunn Nutrition Unit in Cambridge, under the directorship of Dr Roger Whitehead, took over the running of a permanent field station in Keneba. The population of Keneba was now about 900 (Rayco-Solon et al. 2004). To support the research programme and serve the medical needs of the three core villages^1^, a regular outpatient clinic was established. In addition, all children aged between 6 months and 3 years from these villages were brought to the clinic at 4–6 week intervals for routine clinical visits, physical examinations, measurement of anthropometry and clinical assessment (stool and urine microscopy, blood films for malaria, haematology). As the nationwide EPI programme was introduced across The Gambia, childhood vaccines were also given to infants and young children at these clinic visits (Rayco-Solon et al. 2004). From 1976 the survey was extended to include children from birth, with neonatal examinations performed in the child’s home by a trained midwife within 24 hours of birth. From 1977 the clinical care of the three core villages was further extended to include regular antenatal clinics started by a trained nurse-midwife. Pregnant women were seen regularly throughout pregnancy, and deliveries were attended where possible. Dubowitz gestational ageing of all newborns conducted within 5 days of birth by a physician was started in 1978 (Rayco-Solon et al. 2004). By 1985, the Keneba population had increased to approximately 1200. From 1989 the clinic was extended to include a “gate clinic” service, offering primary care to all. All children under 3 years of age were still seen by a doctor. A mobile midwifery service, funded by the WHO Safe Motherhood Initiative, was also initiated, providing antenatal care across West Kiang.

### Phase 3, 2004 to date

Since 2004, the Keneba cohorts have expanded to incorporate a Demographic Surveillance System (DSS), electronic clinic records system and a biobank, each covering the whole population of West Kiang. Full details of each are provided in the paper by Hennig et al. (2017), but brief details are included herewith.

**DSS**. The main purpose of the West Kiang wide DSS is to provide reliable and up-to-date demographic data on the population of West Kiang to support the research portfolio. In addition to capturing all key demographic details and to facilitate longitudinal and follow up studies, it also enables the tracking of individuals’ movements. The current, stable population is ∼14,850 (Hennig et al. 2017). In setting up the DSS, every individual who had been resident in West Kiang since 2004, together with all those individuals no longer resident but who had participated in the research programme before that date, was assigned a new, unique identity number (ID). This allowed full linkage to historical records as well as prospective studies. For data collection, every compound in the district is visited once every 3 months.

**KEMReS**. Established in 2009, the Keneba Electronic Medical Records System (KEMReS) was designed to capture, in real time, clinical data for all presentations at the clinic in Keneba, across all age groups. The database also incorporates data on the regular antenatal, child welfare and vaccination clinics, as described above. In addition to supporting research studies and clinical trials, the collection of clinical data directly has supported a greater review of the epidemiology of communicable and non-communicable diseases, with the broader objective of improving clinical care for the population.

**Keneba biobank**. The Keneba biobank was established in 2012, as a platform for genetic studies through the collection of biological samples and simple phenotypic measures for all consenting individuals captured through the DSS. Sampling and measurements are collected in the field, with visits scheduled to ensure even distributions or recruitment by village and season. Data collected includes information on nutritional status (anthropometry, body composition, haemoglobin), health parameters (blood pressure, fasting blood glucose, full blood count), socio-economic status and additional data on educational attainment and medication use. Collected samples (blood (plasma, serum, washed cells and PBMCs) and urine) are biobanked. The Keneba biobank collection is cross-sectional in nature, with one round of collection completed to date.

This backdrop of long-term demographic record keeping, together with health-related data and, more recently, expanded clinical and phenotypic data for the whole population, has enabled and supported a wealth of nutrition and health related research on this population (Hennig et al. 2017). Below, I describe some key examples of how the data have been used. This is not an exhaustive or systematic presentation of research outputs; papers were selected on the basis that they either used, at the time of analysis, the full extent of data available from each phase of data collection (e.g. Moore et al. 1997, 1999; Rayco-Solon et al. 2004; Jobe et al. 2017; Nabwera et al. 2017; Schoenbuchner et al. 2019) or are some of the best examples of transdisciplinary use of the available data (e.g. Sear et al. 2000; Rickard et al. 2012; Eriksen et al. 2017).

### Demographic data

**Figure 1.**
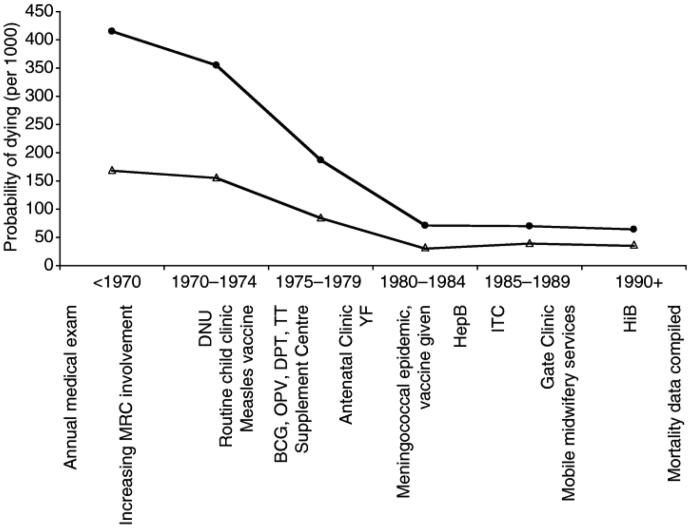
Changes in infant (0–1 year; Δ ) and under‐5 (•) probabilities of dying per 1000 in the three study villages. Major developments are listed at the bottom of the graph. MRC: Medical Research Council Laboratories; DNU: Dunn Nutrition Unit field station established and starts permanent clinic; BCG: Bacille–Calmette–Guerin vaccination started; OPV: oral polio vaccination started; DPT: Diptheria pertusis tetanus vaccination started; TT: tetanus toxoid vaccination started; YF: yellow fever vaccination started; HepB: hepatitis B vaccination started; ITC: International Trypanotolerance Centre established in Keneba; Hib: *Haemophilus influenza* vaccination started. Reproduced with permission.

The maintenance of demographic records over seven decades has allowed a detailed review of secular trends in survival within this context. In a paper published in 2004, Rayco-Solon et al. described trends in neonatal and post-neonatal mortality rates over a period of 50 years (1947–1997), mapped against health interventions (e.g. introduction of childhood vaccination programmes). Over the period studied (pre-1975 to 1990), a dramatic decrease in neonatal (from 44 to 15, per 1000), infant (162 to 36) and under-five mortality rates (367 to 66) was observed, coinciding with the introduction of several health-related interventions (Figure 1).

In response to the growing interest in DOHaD research, the Keneba demographic data have also been used to explore the impact of the early life environment on longer-term health. Using the model of seasonality to define the early life environment, and the demographic data collected between 1949 and 1994, we looked at the impact of season of birth on survival in this context. What we found was unexpected: birth during the annual hungry season (defined as July to December in this analysis) led to a significantly greater risk of premature adult mortality compared with birth during the annual harvest season (January to June) (Moore et al. 1997). Relative to the harvest season, the hazard ratio for early death in hungry-season births rose from 3.7 for deaths over the age of 14.5 years (*p* = .000013) to 10.3 for deaths over the age of 25 years (*p* = .00002). Cause of death was ascertained for over 75% of cases from either field clinic records, hospital records or verbal autopsy from relatives, revealing that the majority of deaths had a definite indication of possible infectious aetiology (Moore et al. 1999); none were from non-communicable diseases, which up to that point had been the primary outcome of focus within the DOHaD field. This finding, suggesting that the human immune system may also be susceptible to early life exposures, led to a programme of work in The Gambia looking at the early life nutritional programming of immunity; a full summary of this programme of research is reviewed in Moore et al. (Moore 2016), with more recent findings published in Moore et al. (2019) and Okala et al. (2019).

### Demographic and health data

The introduction of regular health surveys from the mid-1970s, alongside the maintenance of clinical data collection, has supported a range of research questions. Here, a selected number are presented, chosen to highlight the wide and varied array of approaches that have been taken using the Keneba cohorts data.

Using clinical and anthropometric data collected on all children presenting at the Keneba clinic between 1979 and 1993, Poskitt and Cole (Poskitt et al. 1999) explored whether a reduction in the prevalence of diarrhoea in the community had led to improved growth and a reduced prevalence of malnutrition among children under two years of age. Over the 15-year period of study, there was a significant decrease in both the number of presentations with diarrhoea (from 1,069 in 1979 to 220 in 1993) and in the proportion of clinic attendees presenting with diarrhoea (from 30% in 1979 to 8% in 1993). However, despite these major reductions in clinical presentations with diarrhoea, no associated improvements in nutritional status, assessed by changes in weight and length related z-scores, were observed over this time period, suggesting a broader aetiology to the growth faltering in this area. More recent work by Husseini et al. (2018) almost two decades later (reviewed later in this paper) adds further support for these findings.

**Figure 2.**
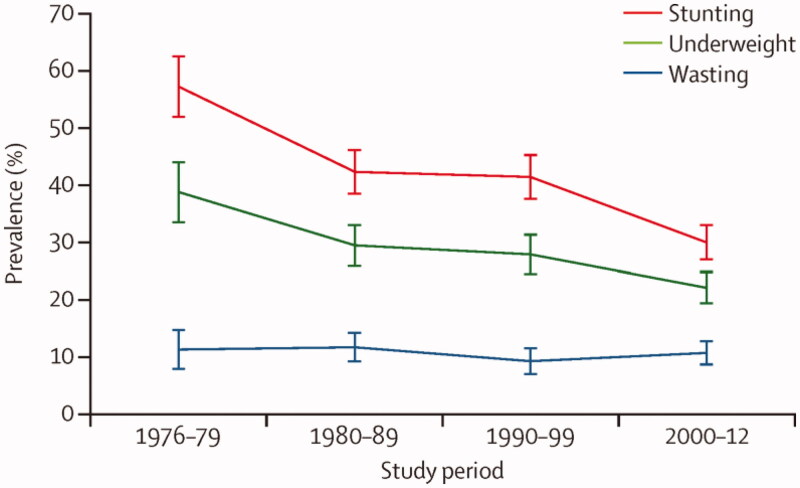
Secular trends in stunting, underweight, and wasting at 2 years of age. Stunting, underweight, and wasting are defined as proportion below −2 *Z* scores against WHO 2006 growth reference standards. Reproduced with permission.

More recently, we have used the routine clinical data collected from 1976 to look at secular trends in the nutritional status of infants and young children from birth to two years of age. Using data on 3,659 children collected between 1976 and 2012, secular improvements were observed in all growth parameters (except weight-for-length) (Nabwera et al. 2017). The proportion of children with underweight or stunting at 2 years of age halved during the four decades of the study period (between the 1970s and 2000s), from 38.7% to 22.1% for underweight and from 57.1% to 30.0% for stunting, (Figure 2). However, despite these impressive declines, what is significant is that rates of underweight and stunting remain high at 22% and 30%, respectively. This is despite substantial and sustained investments by the MRC into health care and nutrition-related infrastructure in this community. The observation of high rates of stunting is consistent with data on global trends, indicating that rates of stunting in Africa have remained high, despite a trend towards reductions across all other regions (de Onis et al. 2012).

The historical demographic records within the Keneba cohorts have also been used to investigate relationships between family structure, infant nutritional status and infant survival, through an anthropological lens. Using records on all children born between 1950 and 1974, where dates of birth and death (where applicable) were known, Sear et al. (2000) found a clear beneficial effect of maternal grandmothers on both child survival and child nutritional status. Furthermore, evidence was also found that the reproductive status of the maternal grandmother influences child nutrition, with young children being taller in the presence on non-reproductive grandmothers than in the presence of grandmothers who are still reproductively active. These relationships were not observed with other grandparents, suggesting that this effect is not the result of an inherited effect but a consequence of some factor specific to the maternal grandmother, such as childcare.

The accumulated demographic and health data have also been used to address two questions in relation to evolutionary and developmental biology. In the first paper, by Rickard et al. (2012), data from Keneba was used to test the hypothesis that maternal twinning status predicts offspring birthweight. This was on a backdrop of evidence to suggest that variations in the dynamics of the insulin-like growth factor (IGF) system – a key regulator of foetal growth – may also influence twinning propensity. Using a cohort of 1889 singleton infants born between 1978 and 2009, for those who were born between January and June and thus were hypothesised to experience a favourable *in utero* environment, births before and after twins were associated with an increase in birth weight (134 and 226 g increase for those born before and after twins, respectively) compared to those born to non-twinning mothers. Of note, these findings were not mediated by maternal body size. An intriguing finding, but highlighted here to demonstrate the wide utility across research domains of the collated longitudinal data contained in the Keneba cohorts.

In a second paper, also related to *in utero* energy exposure, Eriksen et al. (2017) investigated associations between parental energy exposure and nutrient restriction *in utero* and the growth of their own offspring. Using records for infants born between 1972 and 2011, the authors observed a significant effect of maternal season of birth (but not paternal season of birth) on offspring birth length, but only the father’s season of birth was important in offspring length at 2 years of age. These findings are consistent with the hypotheses that foetal growth is under the influence of the maternal line, whereas postnatal growth has intergenerational influences from the paternal line (Eriksen et al. 2017). There are several potential mechanistic routes that could explain these observations, including that the epigenome of the developing parental germline is altered by gestational nutritional status, contributing to altered offspring development. Such hypotheses remain to be tested directly in human populations.

### Enhanced clinical data collection and health research

The introduction of direct electronic data collection through the KEMReS system, alongside the expanded West Kiang DSS and biobank, has enabled a greater depth of research into the health and health care provision of the West Kiang population. Below, the findings from three recent publications are summarised.

Firstly, using data extracted from the KEMReS system, Rees et al. (2016) looked at factors affecting access to healthcare in children under 5 years of age. Using data collected between 2009 and 2012, first clinic attendances with malaria, lower respiratory tract infection and diarrhoeal disease were identified and categorised as delayed/non-delayed and severe/non-severe. This analysis indicated that, even in a context where healthcare is delivered free of charge, availability of transport was identified as the most important barrier to accessing healthcare, supporting the need for public health interventions focussed on improving access to healthcare facilities (Rees et al. 2016).

The KEMReS data have further been used to look at potential interactions between nutritional status and disease severity in children under 5 years of age presenting to the clinic in Keneba. Using data collected during a five-year period (2010–2014) and including data on 21,278 clinic visits with 26,001 diagnoses, it was found that wasting was associated with an increased risk of severe illness in a dose-dependent manner whereas stunting, even in the most severe form (HAZ< −3), was not significantly associated with severe illness but was associated with a significant risk of death (Mark et al. 2019).

Finally, and using clinical data collected as part of the Keneba biobank, Jobe et al. (Jobe et al. 2017) used cross-sectional data on 6160 healthy Gambians aged > 5 years to look at the prevalence of high blood pressure within this rural context. This analysis was in response to the scarcity of similar data from rural sub-Saharan Africans, especially among adolescents and young adults in these contexts. Among generally lean young Gambians (<18 years), a prevalence of high blood pressure was observed in 8.2%. Given that a raised blood pressure in childhood can aggravate cardiovascular outcomes in later life, this finding, enabled by the addition of clinical phenotyping data to the Keneba cohorts, suggests a greater focus on non-communicable diseases may be required within this rural context in the future.

### Integrating research to support nutrition and health policy and planning

More recently, the accumulated data from the Keneba cohorts have been used to answer research questions of direct relevance to nutrition and health policy and planning.

**Figure 3.**
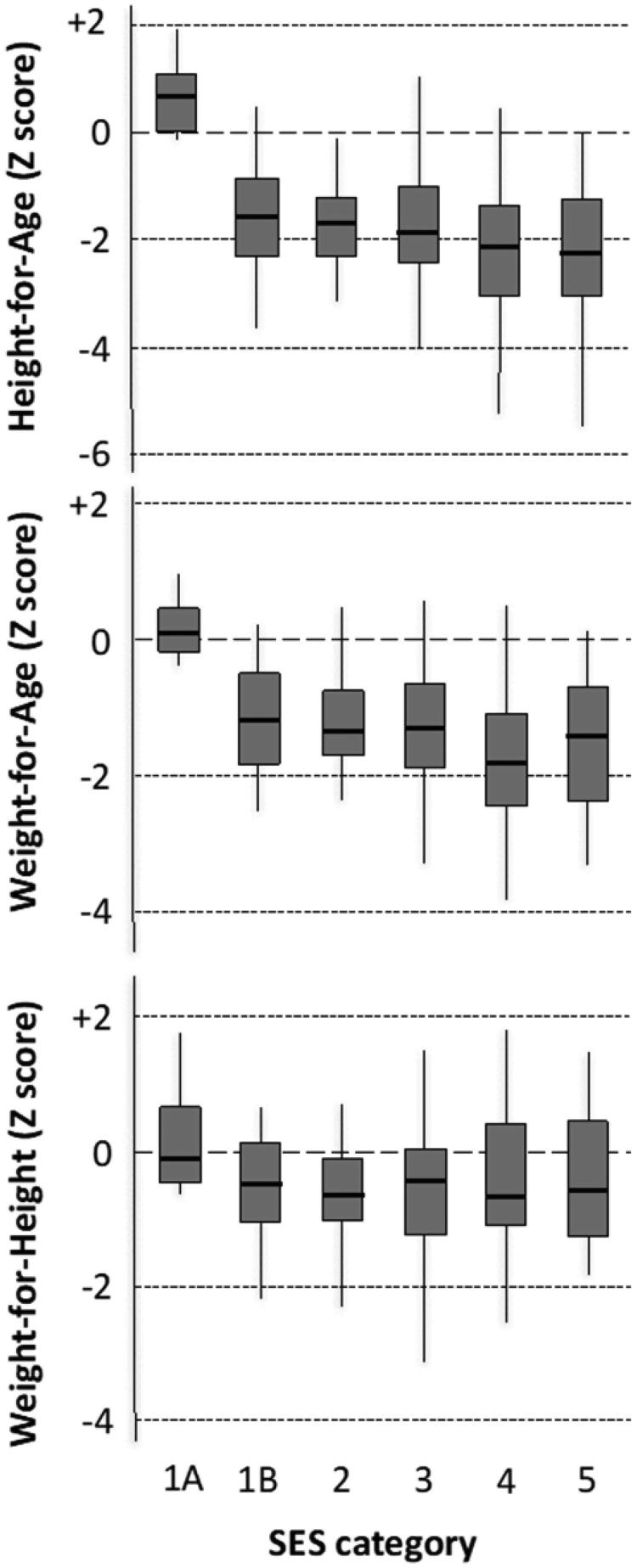
Means, interquartile ranges and 95% confidence intervals for anthropometric scores at 24 m. Reproduced with permission.

Recent emphasis within the nutrition field has focussed on the relevance of nutrition-specific interventions (interventions that address immediate causes of malnutrition, e.g. micronutrient supplementation) versus nutrition-sensitive interventions (interventions in other sections that incorporate nutrition objectives, e.g. water, sanitation and hygiene (WASH) interventions) to improve child growth and development. However, despite recent investments in several large intensive nutrition-sensitive interventions, the impact on childhood growth has been disappointingly small (Pickering et al. 2019). Using nutritional status and socioeconomic status (SES) data from the Keneba setting, we explored the thresholds of SES and living standards associated with childhood undernutrition. A composite SES score was generated by grading occupation, education, income, water and sanitation and housing, and families were allocated to 5 groups (SES1 = highest). The findings from this analysis indicated a very high SES threshold before stunting and underweight were reduced (Husseini et al. 2018). A shallow gradient was observed in child nutritional status across all groups (Figure 3) from SES5 to SES1B. Only the children from the very highest group (SES1A) – who were children of MRC staff living on the MRC camp in Western-style housing – showed good growth – all other groups were virtually indistinguishable.

The accumulated data of infant growth from the Keneba cohorts reveals a growth pattern very typical of children from resource poor settings in both Africa and Southeast Asia, with a rapid falloff in HAZ during the first 2 years of life and no recovery until after five years of age (Victora et al. 2010; Prentice et al. 2013). This observed pattern of growth underpins the recent focus on the period from conception to two years of age (coined the first 1000 days) and there has been a recent investment and focus on interventions targeting this period. We have used the longitudinal growth data from the Keneba cohorts to argue that – whilst investments in the first 1000 days should not be undermined – there are also other periods across the life-course where investments in nutrition and health interventions may be effective. To illustrate this point, the longitudinal growth data from rural Gambia demonstrates an extended pubertal growth phase, allowing considerable height recovery, especially in girls during adolescence (Prentice et al. 2013). These data have contributed towards the argument that adolescence represents an additional window of opportunity during which substantial life cycle and intergenerational effects can be accrued.

The same longitudinal data have also been used in a recent study into the relationship between wasting and stunting. To provide context, the background to this investigation was the growing concern within the nutrition community that the separate management (both clinically and programmatically) of stunting and wasting may not be beneficial to treatment of prevention programmes. Further, a closer investigation of the aetiological relationship between wasting and stunting is required to help improve our understanding of how best to manage these two outcomes of undernutrition (Briend et al. 2015). One of the barriers to understanding this relationship has been the lack of high-quality longitudinal data from children at risk of undernutrition. Using the anthropometric data collected at the scheduled infant welfare clinics since the mid-1970s, we looked at the interrelationships between wasting and stunting in children aged < 2 years (Schoenbuchner et al. 2019). Consistent with previous research, levels of both wasting and stunting were observed to be high, peaking at approximately (girls–boys) 12–18% at 10 to 12 months (wasting) and 37–39% at 24 months (stunting). Boys were also more likely to be both wasted and stunted and to be concurrently wasted and stunted. By modelling growth according to season of birth, we also observed that infants born at the start of the annual wet season (born in July) showed early growth faltering in weight-for-length z-score and were at increased risk of subsequent stunting. Finally, using time-lagged observations, we demonstrated that being wasted was predictive of subsequent stunting (Odds Ratio: 3.2; 95% Confidence Interval: 2.7, 3.9), even after accounting for current stunting (Schoenbuchner et al. 2019). This analysis supports previous arguments that stunting is, in part, a biological response to previous episodes of being wasted and is important from a policy perspective as it suggests greater emphasis should be put on aligning the management of these two outcomes of undernutrition (Wells et al. 2019).

## Conclusions and implications

The research highlighted in this review represents a selection of some of the outputs that have been possible because of the vision of the late Professor Sir Ian McGregor to initiate demographic data collection some seven decades ago, coupled with long-term investment into data collection by the UK MRC. The resource available from the Keneba cohorts represents one of the most detailed longitudinal collections of data – and latterly biological samples – from a low- and middle-income country and is the most detailed collection for a rural population in sub-Saharan Africa. Together, the wealth of demographic and health data has enabled a variety of important insights into many aspects of human health, more latterly contributing important data for nutrition programming and policy. The example of the Keneba cohorts underpins the importance of cohort data for research into human biology and health and emphasises the importance of having longitudinal cohorts across population groups.

## Note

1 The village of Jali selected not to participate in the enhanced demographic and health surveillance, but remained engaged in research studies and the Phase 3 process of data collection.

## References

[CIT0001] Briend A, Khara T, Dolan C. 2015. Wasting and stunting--similarities and differences: policy and programmatic implications. Food Nutr Bull. 36(1_suppl1):S15–S23. 25902610 10.1177/15648265150361S103

[CIT0002] de Onis M, Blossner M, Borghi E. 2012. Prevalence and trends of stunting among pre-school children, 1990-2020. Public Health Nutr. 15(1):142–148. 21752311 10.1017/S1368980011001315

[CIT0003] Elliott J, Shepherd P. 2006. Cohort profile: 1970 British Birth Cohort (BCS70). Int J Epidemiol. 35(4):836–843. 16931528 10.1093/ije/dyl174

[CIT0004] Eriksen KG, Radford EJ, Silver MJ, Fulford AJC, Wegmuller R, Prentice AM. 2017. Influence of intergenerational in utero parental energy and nutrient restriction on offspring growth in rural Gambia. Faseb J. 31(11):4928–4934. 28778976 10.1096/fj.201700017R PMC5636699

[CIT0005] Fall CH, Sachdev HS, Osmond C, Restrepo-Mendez MC, Victora C, Martorell R, Stein AD, et al. 2015. Association between maternal age at childbirth and child and adult outcomes in the offspring: a prospective study in five low-income and middle-income countries (COHORTS collaboration). Lancet Glob Health. 3:e366–77. 25999096 10.1016/S2214-109X(15)00038-8 PMC4547329

[CIT0006] Fraser A, Macdonald-Wallis C, Tilling K, Boyd A, Golding J, Smith GD, Henderson J, et al. 2013. Cohort Profile: the Avon Longitudinal Study of Parents and Children: ALSPAC mothers cohort. Int J Epidemiol. 42(1):97–110. 22507742 10.1093/ije/dys066 PMC3600619

[CIT0007] Hanson M. 2015. The birth and future health of DOHaD. J Dev Orig Health Dis. 6(5):434–437. 26004094 10.1017/S2040174415001129

[CIT0008] Hennig BJ, Unger SA, Dondeh BL, Hassan J, Hawkesworth S, Jarjou L, Jones KS, et al. 2017. Cohort Profile: The Kiang West Longitudinal Population Study (KWLPS)-a platform for integrated research and health care provision in rural Gambia. Int J Epidemiol. 46(2):e13. 26559544 10.1093/ije/dyv206 PMC5837564

[CIT0009] Husseini M, Darboe MK, Moore SE, Nabwera HM, Prentice AM. 2018. Thresholds of socio-economic and environmental conditions necessary to escape from childhood malnutrition: a natural experiment in rural Gambia. BMC Med. 16(1):199. 30382849 10.1186/s12916-018-1179-3 PMC6211595

[CIT0010] Jobe M, Agbla SC, Prentice AM, Hennig BJ. 2017. High blood pressure and associated risk factors as indicator of preclinical hypertension in rural West Africa: a focus on children and adolescents in The Gambia. Medicine. 96(13):e6170. 28353557 10.1097/MD.0000000000006170 PMC5380241

[CIT0011] Mark H, Been JV, Sonko B, Faal A, Ngum M, Hasan J, Prentice AM, Unger SA. 2019. Nutritional status and disease severity in children acutely presenting to a primary health clinic in rural Gambia. BMC Public Health. 19(1):668. 31146716 10.1186/s12889-019-6959-y PMC6543667

[CIT0012] Martorell R, Horta BL, Adair LS, Stein AD, Richter L, Fall CH, Bhargava SK, et al. 2010. Weight gain in the first two years of life is an important predictor of schooling outcomes in pooled analyses from five birth cohorts from low- and middle-income countries. J Nutr. 140:348–354. 20007336 10.3945/jn.109.112300 PMC2806888

[CIT0013] McGregor IA. 1985. Seroepidemiological studies of malaria in The Gambia, West Africa. Ann Soc Belg Med Trop. 65 Suppl 2(Suppl 2):79–87. 3907544

[CIT0014] McGregor IA, Wilson ME, Billewicz WZ. 1983. Malaria infection of the placenta in The Gambia, West Africa; its incidence and relationship to stillbirth, birthweight and placental weight. Trans R Soc Trop Med Hyg. 77(2):232–244. 6346592 10.1016/0035-9203(83)90081-0

[CIT0015] Moore SE. 2016. Early life nutritional programming of health and disease in The Gambia. J Dev Orig Health Dis. 7(2):123–131. 26503192 10.1017/S2040174415007199 PMC4825101

[CIT0016] Moore SE, Cole TJ, Collinson AC, Poskitt EM, McGregor IA, Prentice AM. 1999. Prenatal or early postnatal events predict infectious deaths in young adulthood in rural Africa. Int J Epidemiol. 28(6):1088–1095. 10661652 10.1093/ije/28.6.1088

[CIT0017] Moore SE, Cole TJ, Poskitt EM, Sonko BJ, Whitehead RG, McGregor IA, Prentice AM. 1997. Season of birth predicts mortality in rural Gambia. Nature. 388(6641):434–434. 9242401 10.1038/41245

[CIT0018] Moore SE, Fulford AJC, Sosseh F, Nshe P, Darboe MK, Prentice AM. 2019. Thymic size is increased by infancy, but not pregnancy, nutritional supplementation in rural Gambian children: a randomized clinical trial. BMC Med. 17(1):38. 30773140 10.1186/s12916-019-1264-2 PMC6378709

[CIT0019] Nabwera HM, Fulford AJ, Moore SE, Prentice AM. 2017. Growth faltering in rural Gambian children after four decades of interventions: a retrospective cohort study. Lancet Glob Health. 5(2):e208–e216. 28104187 10.1016/S2214-109X(16)30355-2 PMC5340725

[CIT0020] Norris SA, Daar A, Balasubramanian D, Byass P, Kimani-Murage E, Macnab A, Pauw C, et al. 2017. Understanding and acting on the developmental origins of health and disease in Africa would improve health across generations. Glob Health Action. 10:1334985. 28715931 10.1080/16549716.2017.1334985 PMC5533158

[CIT0021] Okala SG, Darboe MK, Sosseh F, Sonko B, Faye-Joof T, Prentice AM, Moore SE. 2019. Impact of nutritional supplementation during pregnancy on antibody responses to diphtheria-tetanus-pertussis vaccination in infants: a randomised trial in The Gambia. PLoS Med. 16(8):e1002854. 31386660 10.1371/journal.pmed.1002854 PMC6684039

[CIT0022] Pickering AJ, Null C, Winch PJ, Mangwadu G, Arnold BF, Prendergast AJ, Njenga SM, et al. 2019. The WASH Benefits and SHINE trials: interpretation of WASH intervention effects on linear growth and diarrhoea. Lancet Glob Health. 7(8):e1139–e1146. 31303300 10.1016/S2214-109X(19)30268-2

[CIT0023] Poskitt EM, Cole TJ, Whitehead RG. 1999. Less diarrhoea but no change in growth: 15 years' data from three Gambian villages. Arch Dis Child. 80(2):115–119. 10325724 10.1136/adc.80.2.115 PMC1717825

[CIT0024] Power C, Elliott J. 2006. Cohort profile: 1958 British birth cohort (National Child Development Study). Int J Epidemiol. 35(1):34–41. 16155052 10.1093/ije/dyi183

[CIT0025] Prentice AM, Moore SE, Fulford AJ. 2013. Growth faltering in low-income countries. World Rev Nutr Diet. 106:90–99. 23428686 10.1159/000342563

[CIT0026] Prentice AM, Ward KA, Goldberg GR, Jarjou LM, Moore SE, Fulford AJ, Prentice A. 2013. Critical windows for nutritional interventions against stunting. Am J Clin Nutr. 97(5):911–918. 23553163 10.3945/ajcn.112.052332 PMC3628381

[CIT0027] Rayco-Solon P, Moore SE, Fulford AJ, Prentice AM. 2004. Fifty-year mortality trends in three rural African villages. Trop Med Int Health. 9(11):1151–1160. 15548310 10.1111/j.1365-3156.2004.01325.x

[CIT0028] Rees CP, Hawkesworth S, Moore SE, Dondeh BL, Unger SA. 2016. Factors affecting access to healthcare: an observational Study of children under 5 years of age presenting to a Rural Gambian Primary Healthcare Centre. PLoS One. 11(6):e0157790. 27336164 10.1371/journal.pone.0157790 PMC4919103

[CIT0029] Richter LM, Victora CG, Hallal PC, Adair LS, Bhargava SK, Fall CH, Lee N, the COHORTS Group, et al. 2012. Cohort profile: the consortium of health-orientated research in transitioning societies. Int J Epidemiol. 41(3):621–626. 21224276 10.1093/ije/dyq251 PMC3378468

[CIT0030] Rickard IJ, Prentice AM, Fulford AJ, Lummaa V. 2012. Twinning propensity and offspring in utero growth covary in rural African women. Biol Lett. 8(1):67–70. 21831878 10.1098/rsbl.2011.0598 PMC3259965

[CIT0031] Schoenbuchner SM, Dolan C, Mwangome M, Hall A, Richard SA, Wells JC, Khara T, et al. 2019. The relationship between wasting and stunting: a retrospective cohort analysis of longitudinal data in Gambian children from 1976 to. Am J Clin Nutr. 110(2):498–507. 30753251 10.1093/ajcn/nqy326 PMC6669055

[CIT0032] Sear R, Mace R, McGregor IA. 2000. Maternal grandmothers improve nutritional status and survival of children in rural Gambia. Proc Biol Sci. 267(1453):1641–1647. 11467427 10.1098/rspb.2000.1190 PMC1690719

[CIT0033] Stein AD, Wang M, Martorell R, Norris SA, Adair LS, Bas I, Sachdev HS, On Behalf of the Cohorts Group, et al. 2010. Growth patterns in early childhood and final attained stature: data from five birth cohorts from low- and middle-income countries. Am J Hum Biol. 22(3):353–359. 19856426 10.1002/ajhb.20998 PMC3494846

[CIT0034] Syddall HE, Aihie Sayer A, Dennison EM, Martin HJ, Barker DJ, Cooper C. 2005. Cohort profile: the Hertfordshire cohort study. Int J Epidemiol. 34(6):1234–1242. 15964908 10.1093/ije/dyi127

[CIT0035] Victora CG, de Onis M, Hallal PC, Blossner M, Shrimpton R. 2010. Worldwide timing of growth faltering: revisiting implications for interventions. Pediatrics. 125(3):e473–80. 20156903 10.1542/peds.2009-1519

[CIT0036] Wells JCK, Briend A, Boyd EM, Berkely JA, Hall A, Isanaka S, Webb P, et al. 2019. Beyond wasted and stunted-a major shift to fight child undernutrition. Lancet Child Adolesc Health. 3:831–834. 31521500 10.1016/S2352-4642(19)30244-5

[CIT0037] Wright J, Small N, Raynor P, Tuffnell D, Bhopal R, Cameron N, Fairley L, Born in Bradford Scientific Collaborators Group, et al. 2013. Cohort Profile: the Born in Bradford multi-ethnic family cohort study. Int J Epidemiol. 42(4):978–991. 23064411 10.1093/ije/dys112

